# A Complex Chromosome Rearrangement Disrupting *SYT1* Supports Haploinsufficiency as a Cause of Baker–Gordon Syndrome

**DOI:** 10.1155/crig/6652420

**Published:** 2025-12-15

**Authors:** Débora Romeo Bertola, Sofia de Oliveira Farias, Silvia Souza da Costa, Mara Maria Lisboa Santana Pinheiro, Maria Rita dos Santos Passos-Bueno, Carla Rosenberg, Ana Cristina Victorino Krepischi

**Affiliations:** ^1^ Institute of Biosciences, Department of Genetics and Evolutionary Biology, Human Genome and Stem Cell Research Center, University of Sao Paulo, São Paulo, Brazil, usp.br; ^2^ Unidade de Genética, Instituto da Criança, Hospital das Clínicas da Faculdade de Medicina, Universidade de São Paulo, São Paulo, Brazil, usp.br

**Keywords:** BAGOS, chromosomal rearrangement, OGM, SYT1

## Abstract

Baker–Gordon syndrome (BAGOS) is an autosomal dominant neurodevelopmental disorder caused by *de novo* heterozygous missense mutations in *SYT1*. The precise pathogenic mechanism of BAGOS is still unclear, with preliminary data favoring a dominant‐negative effect, although a previous case presenting a reciprocal translocation disrupting *SYT1* supports haploinsufficiency as a possible mechanism. We report a child with a syndromic neurodevelopmental disorder compatible with BAGOS and carrying a t(5; 12)(q31; q21) by G‐banded karyotype. Optical genome mapping (OGM) is based on ultrahigh molecular weight DNA molecules allowing the combined analyses of numerical and structural chromosome variants. The rearrangement was investigated using OGM, which revealed an additional structural variant, a paracentric inversion in the segment of Chromosome 12 translocated to der(5). The breakpoint of the paracentric inversion is mapped to Intron 9 of the *SYT1* gene, interrupting the C2B domain. This is the second BAGOS case reported in the literature caused by *SYT1* disruption, supporting that reduced amounts of functional SYT1, either by haploinsufficiency or dominant‐negative effect, is responsible for *SYT1*‐associated neurodevelopmental syndrome.

## 1. Introduction

Baker–Gordon syndrome (BAGOS—OMIM #618218) is an autosomal dominant neurodevelopmental disorder caused by *SYT1* (OMIM ^∗^185605) mutations and characterized by developmental delay and intellectual disability, poor or absent speech, behavioral psychiatric manifestations, moderate to severe sleep disturbances, electroencephalogram (EEG) abnormalities, movement disorders, and abnormal eye physiology [1, 2]. Although the molecular mechanism of BAGOS is not fully understood, the large majority of cases are caused by *de novo* heterozygous missense variants in the C2B domain of *SYT1* [1, 2]. Data of impaired synaptic transmission in neurons expressing these mutations indicate that dominant‐negative effect is likely the molecular basis of *SYT1*‐associated neurodevelopmental disorder [3]. Nonetheless, a recent case was reported presenting a reciprocal translocation between Chromosomes 7 and 12, with one of the breakpoints mapped in the second intron of *SYT1* [4], supporting haploinsufficiency as the molecular mechanism responsible for the neurodevelopmental features for this patient.

## 2. Methods

This study was approved by the research ethics committee of the Institute of Bioscience of University of São Paulo. Written informed consent was obtained from the mother of the patient; the father was unavailable.

G‐banded karyotype from peripheral blood samples was performed according to the standard procedure. Fluorescence *in situ* hybridization (FISH) analysis based on metaphase spreads and interphase preparations was performed as previously described [5], using BAC clones flanking the *SYT1* gene: RP11‐26L7 (chr12: 78057303‐78227311‐hg38; labeled using the digoxigenin/rhodamine system) and RP11‐44H8 (chr12: 79662585‐79828021‐hg38; labeled with biotin/avidin FITC system), both mapped to the 12q21.2 cytoband.

Chromosome microarray analysis (array‐CGH) was done according to the manufacturer’s instructions, using a 180K oligonucleotide platform (Agilent Technologies). Data were extracted and analyzed for copy number changes using the software Nexus Copy Number Discovery (Bionano), as previously reported [6].

For further characterization of the detected chromosomal translocation, the OGM analysis was performed using ultrahigh molecular weight DNA samples extracted from peripheral blood cells (Bionano). DNA samples were labeled with the DLS DNA labeling kit to add fluorophores to the CTTAAG motif (DLE1 enzyme). The Saphyr System was used at 100 × coverage, and OGM data were analyzed after *de novo* assembly using both CNV and SV pipelines. Visualization and analyses were performed using the Bionano Access v8 software. Genomic coordinates are described according to GRCh38.

## 3. Results

### 3.1. Clinical Description

The proband is a 12‐year‐old girl, the only child of nonconsanguineous, young parents. She was born at term, after an uneventful pregnancy, weight of 2660 g, length of 45 cm and occipitofrontal circumference (OFC) of 33 cm. The patient required phototherapy for jaundice and had some feeding difficulties due to poor sucking. She was a quiet, sleepy baby, who evolved with recurrent urinary infections, with a diagnosis of Grade III/IV vesicoureteral reflux, requiring prophylactic antibiotic therapy. She had normal ophthalmological and endocrinologic evaluations, the latter due to excessive body hair.

Her developmental milestones were delayed: She walked independently at 2 years of age and showed severe speech impairment. Intellectual disability and autism spectrum disorder were diagnosed. Three episodes of nonfebrile tonic/tonic‐clonic seizures were documented, the first one at 7 years of age. EEG studies and cranial magnetic resonance imaging (MRI) did not disclose abnormalities. The medication with carbamazepine presented a good response. The patient also exhibited severe behavior abnormalities with hetero/autoaggression, alternating calm and agitated phases, as well as sleep disturbance. Antipsychotic medications were prescribed with poor control of the mood swing and agitation.

Physical examination at the age of 11 years and 8 months showed weight of 36.15 kg (10th centile), height of 133 cm (< 5th centile), OFC of 53.5 cm (50th centile), thick hair, upslanting palpebral fissures, synophrys, anteverted nares, thick lips, and hypertrichosis. She exhibited typical female genitalia, with B2P4 pubertal Tanner stage.

Previous investigation by exome sequencing had disclosed a heterozygous missense variant in *IVD* (NM_001609‐4): c358G > A: p.Gly120Arg, associated with an autosomal recessive inborn error of leucine metabolism (OMIM #243500). Biochemical studies including urinary organic acids and acylcarnitine profile analyses were normal.

### 3.2. Cytogenetic and Molecular Results

Genetic investigation disclosed a G‐banded karyotype with a reciprocal balanced translocation [46,XX,t(5,12)(q31;q21)], not maternal (father not available). Chromosomal microarray analyses did not identify clinically relevant CNVs.

The OGM analysis revealed a four breakpoints rearrangement. In addition to the balanced translocation t(5,12) (Figure 1(a)) detected by karyotype, it was detected a paracentric inversion in the translocated segment of Chromosome 12 (Figure 1(b)), mapped at only 11 kb of the translocation breakpoint at der(5): ogm[GRCh38] (X,1–22) × 2, t(5,12)(q21.3; q14.2)(107673586; 63775716), inv(12)(q14.2q21.1)(63786531_79358882).

Figure 1OGM detects the translocation t(5;12) and reveals an associated inversion disrupting *SYT1*. (a) Translocation t(5;12): segments of the reference chromosomes are represented by gray bars, above Chromosome 12 (Ref 12) and below Chromosome 5 (Ref 5). The vertical lines in the gray bars depict the normal labeling patterns for the regions. The hybrid molecule (light blue bar) shows the breakpoints and junction regions (purple) of the reciprocal translocation t(5;12). These genomic breakpoints were mapped on der(5) and der(12), potentially affecting the sequences of EFNA5 and RXYLT1 genes, respectively. (b) Paracentric inversion: On the left, the image depicts an overview of the paracentric inversion, Chromosome 12 sequences are represented in dark blue, and Chromosome 5 in yellow; the hourglass represents the inverted region of Chromosome 12. On the right, the segment of the reference Chromosome 12 is represented by a gray bar, and the vertical lines in the gray bar depict the normal labeling pattern for the region. The hybrid molecule (light blue bar) shows the proximal breakpoint and junction region, which occurred within the segment of Chromosome 12 translocated to der(5). This detailed view of the proximal region of the inversion (brown bar) shows that this breakpoint affects Intron 9 of *SYT1* (yellow bar).

**Figure:**
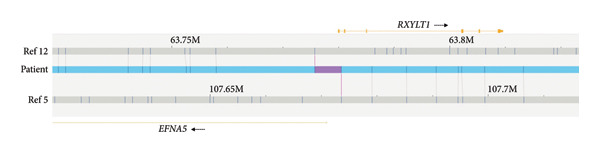
(a)

**Figure:**
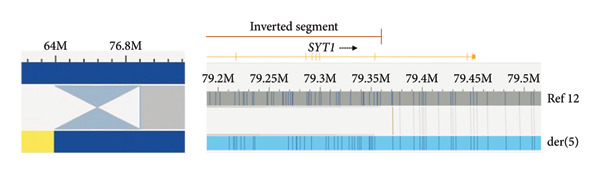
(b)

Analysis of the translocation breakpoint sequence at Chromosome 5 (chr5:107666585–107673586‐hg38) showed that the maximum region includes part of the *EFNA5* (OMIM ^∗^601535) sequence, while the breakpoint at Chromosome 12 (chr12:63775716–63786531‐hg38) affects the *RXYLT1* gene (OMIM ^∗^605862). In the inversion, the proximal region (chr12:63775716–63786531‐hg38) also includes sequences of the *RXYLT1* gene, and, notably, the distal one (chr12:79358882–79371001‐hg38) disrupts *SYT1* (Intron 9; NM_005639.3). FISH analyses with sequences flanking *SYT1* confirmed the paracentric inversion (Figure 2(a)). The scheme of the rearrangement is shown in Figure 2(b).

Figure 2FISH analysis and scheme of the rearrangement. (a) FISH on metaphase chromosomes of the patient using BAC clones flanking the *SYT1* gene: RP11‐26L7 (red) and RP11‐44H8 (green), both mapped at 12q21.2 cytoband. The image shows colocalization of the red and green signals on Chromosome 12; on the der(5) within the translocated segment of Chromosome 12, a gap between red and green signals is observed. The distance between the two signals corroborates the paracentric inversion on der(5) detected by OGM. (b) Complex rearrangement involving a reciprocal t(5;12) and an inv(12)(q14.2q21.1). The G‐banded chromosome pairs involved in the rearrangement are shown: In each pair, the rearranged homologs are at the right, and their normal homologs are at the left. In the middle of the figure, ideograms of Chromosomes 5 (green) and 12 (red) and their translocated homologs (red/green chromosomes)—arrows show the breakpoints of the paracentric inversion, revealed by OGM.

**Figure:**
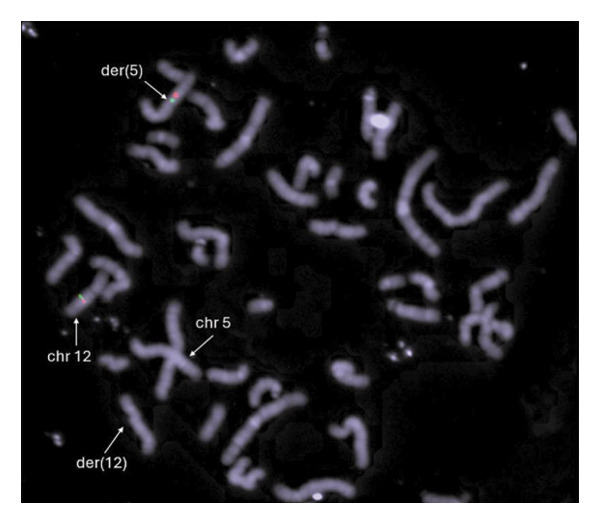
(a)

**Figure:**
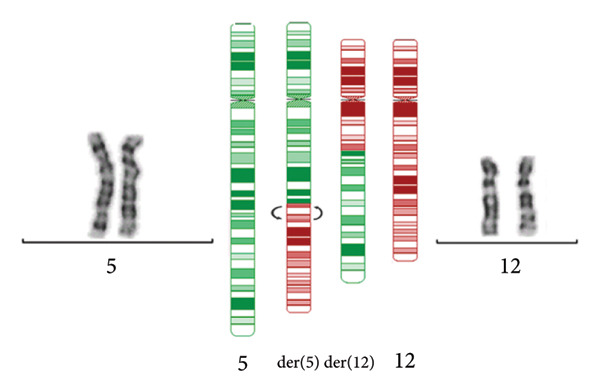
(b)

## 4. Discussion

Here, we describe clinical and molecular features of an individual presenting severe neurodevelopmental disorder, who harbors a chromosome rearrangement that was analyzed by cytogenomic methods. The use of OGM allowed to map the breakpoints of a balanced translocation and to reveal a paracentric inversion, disclosing the disruption of *SYT1.* These results highlight the effectiveness of this technology in revealing complex structural variants.

In complex rearrangements, like the one reported here, the genotype–phenotype correlation is not always straightforward. Other genes were involved in the rearrangement, namely, *EFNA5* and *RXYLT1*. *EFNA5* is an ephrin ligand not known to be associated with a Mendelian phenotype; mice knockout studies suggest a role in ocular lens development and synaptogenic events [7]. *RXYLT1* is associated with muscular dystrophy‐dystroglycanopathy (congenital with brain and eye anomalies), Type A, 10 (OMIM #615041), which is a recessive disorder with clinical features not exhibited by the patient. From the data obtained, there is no evidence of fusion genes, although we cannot exclude that using techniques with different resolution, such as sequencing, fusion genes would be present. However, that is not likely since the patient’s phenotype is consistent with BAGOS which, on the other hand, is explained by the cytogenetic findings.

Among the sequences possibly involved in the chromosomal rearrangement, *SYT1* is the gene that presents clear genomic disruption, along with a dominant Mendelian phenotype (BAGOS) compatible with the clinical picture presented by the proband. The cardinal features of BAGOS include hypotonia, developmental delay, poor/absent speech, movement disorders, and behavioral and eye anomalies. Although a high rate of abnormal electrophysiological pattern on EEG studies has been identified, only 7 individuals have been reported with seizures, particularly absent ones (6/7) starting in childhood [2, 8–10]. Our proband presented a less commonly described type in this neurodevelopmental disorder, tonic‐clonic type seizures, well controlled with antiepileptic drug, also starting in childhood. As BAGOS is a recently reported neurodevelopmental disorder, with less than 40 individuals described in the literature, the precise frequency and severity of each sign/symptom is still not well‐defined. Nevertheless, the phenotypic severity observed in the case described here is similar to patients reported in the literature, harboring either missense or inframe variants. The developmental milestones achieved in the present case are compatible with those described for BAGOS, particularly for the intellectual disability, behavior problems, and severe speech impairment. Additional reports, with long‐term follow‐up, are required to delineate the full‐blown phenotype of this neurodevelopmental disorder.

BAGOS is an autosomal dominant neurodevelopmental disorder caused by *de novo* mutations in *SYT1*. *SYT1* codes for a membrane protein that plays a role in vesicular trafficking and exocytosis [11, 12]. It has a significant role in the physiology of synaptic neurotransmission, and the impairment of this protein can result in severe neurological disorders. The precise pathogenic mechanism of *SYT1*‐associated neurodevelopmental disorders is still unclear, with preliminary data favoring a dominant‐negative effect [3, 10].

The breakpoint disrupting *SYT1* in the present case maps to Intron 9, abolishing the C2B domain, where most of the pathogenic missense variants linked to *SYT1*‐associated neurodevelopmental disorders are located, probably interfering with calcium‐binding properties. Thus, this gene has been assigned as the one likely responsible for the severe neurodevelopmental phenotype in the present case.

A single case of BAGOS phenotype also associated with a reciprocal translocation disrupting *SYT1* has been previously reported [4].

## 5. Conclusions

To our knowledge, this is the second individual presenting BAGOS caused by *SYT1* disruption, supporting the hypothesis that reduced amounts of functional SYT1, either by haploinsufficiency or dominant‐negative effect, is responsible for *SYT1*‐associated neurodevelopmental disorders.

## Conflicts of Interest

The authors declare no conflicts of interest.

## Funding

The work was supported by FAPESP, 2013/08028‐1, 2024/07932‐0 and CNPq, 303375/2019‐1.

## Data Availability

The data that support the findings of this study are available on request from the corresponding author. The data are not publicly available due to privacy or ethical restrictions.
